# Some mice lacking intrinsic, as well as death receptor induced apoptosis and necroptosis, can survive to adulthood

**DOI:** 10.1038/s41419-022-04731-x

**Published:** 2022-04-07

**Authors:** Francine F. S. Ke, Kerstin Brinkmann, Anne K. Voss, Andreas Strasser

**Affiliations:** 1 The Walter and Eliza Hall Institute of Medical Research, Melbourne, VIC Australia; 2 The Department of Medical Biology, Melbourne University, Melbourne, VIC Australia

**Keywords:** Developmental biology, Cell biology, Medical research

## Abstract

Programmed cell death, in particular the intrinsic apoptotic pathway, has been shown to play a critical role in the shaping of tissues during embryonic development. The multi-BCL-2 Homology (BH) domain effectors of apoptosis, BAX, BAK, and BOK, are essential for cell killing in the intrinsic apoptotic pathway. It was therefore surprising that we found earlier that a few mice lacking all effectors of apoptosis (*Bax;Bak;Bok* triple knockout), albeit many fewer than expected based on Mendelian ratios, could reach weaning or even adulthood. This indicated that death receptor induced apoptosis or necroptosis, a lytic form of programmed cell death, may also have roles in embryogenesis alongside the intrinsic apoptotic pathway. To explore this, we generated *Bax;Bak;Bok;caspase-8;Mlkl* quintuple knockout mice, which lack not only intrinsic apoptosis but also death receptor induced apoptosis (loss of caspase-8) and necroptosis (loss of MLKL). These foetuses exhibited similar defects to the *Bax;Bak;Bok* triple knockout mice and, intriguingly, a small number of *Bax;Bak;Bok;caspase-8;Mlkl* quintuple knockout mice could reach weaning or even adulthood. These findings identify the contributions of these three programmed cell death pathways to embryonic development and show that despite the absence of all of them, development to adulthood is possible, albeit very rare.

## Introduction

Programmed cell death plays a critical role in embryonic development by removing cells that are no longer needed, damaged, or infected [1]*.* There are several distinct pathways to programmed cell death, including apoptosis, which can be activated through the intrinsic (aka mitochondrial or BCL-2 regulated) or the death receptor induced (aka extrinsic) pathway [2], necroptosis, which can be triggered through activation of death receptors when caspase-8 that is essential for extrinsic apoptosis is absent, or pyroptosis, which can be activated by signals from diverse pathogens and requires caspases-1 or -11 and gasdermin D [3]. The intrinsic apoptotic pathway is regulated by the BCL-2 protein family [2]. In healthy cells, pro-survival BCL-2 family members, such as BCL-2, BCL-XL, and MCL-1, restrain the effectors of cell death, BAK and BAK. Stress conditions, such as nutrient deprivation or anoikis (cell detachment) enhance transcription or cause a post-transcriptional increase of the pro-apoptotic BH3-only proteins (e.g., BIM, PUMA). These critical initiators of apoptosis bind with high affinity to and inhibit the pro-survival BCL-2 proteins, thereby unleashing BAX and BAK to cause mitochondrial outer membrane permeabilisation (MOMP), the point-of-no return in apoptosis signalling. MOMP allows the release of apoptogenic factors (e.g., cytochrome c, SMAC/DIABLO) from the inter-mitochondrial membrane space into the cytoplasm and this leads to the activation of the cascade of caspases that cause the ordered dismantling of the cell [1, 2]. BOK structurally resembles BAX and BAK and can also cause MOMP and apoptosis, but it is neither restrained by the pro-survival BCL-2 proteins nor activated by BH3-only proteins [4, 5].

The intrinsic apoptotic pathway has long been thought to be the major process of programmed cell death that is critical for embryogenesis [6]. Morphological and histological analyses of *Bax*^*−/−*^*Bak*^*−/−*^ and *Bax*^*−/−*^*Bak*^*−/−*^*Bok*^*−/−*^ embryos and mice identified those developmental processes that require apoptosis to occur normally. Common abnormalities seen in E18.5 *Bax*^*−/−*^*Bak*^*−/−*^ and *Bax*^*−/−*^*Bak*^*−/−*^*Bok*^*−/−*^ foetuses include cleft palate/cleft face, aortic arch defects, omphalocele and curled fingers, toes, and tail [5]. Surprisingly, however, many tissues that were thought to depend on apoptosis for development appeared normal in E18.5 *Bax*^*−/−*^*Bak*^*−/−*^ and *Bax*^*−/−*^*Bak*^*−/−*^*Bok*^*−/−*^ foetuses and some of these animals reached the age of weaning or even early adulthood [5, 6]. This raised the possibility that additional programmed cell death pathways, in particular death receptor induced apoptosis and/or necroptosis, might play a role in embryogenesis alongside intrinsic apoptosis. In so-called type 2 cells, death receptor induced apoptosis requires BAX and BAK and would thus be blocked in cells from *Bax*^*−/−*^*Bak*^*−/−*^*Bok*^*−/−*^ embryos [7]. However, in so called type 1 cells death receptor induced activation of caspase-8 with consequent activation of the effector caspases suffices for cell killing with no need for engagement of the intrinsic apoptotic pathway by caspase-8 mediated activation of the pro-apoptotic BH3-only protein BID [8]. Thus, death receptor induced apoptosis would be possible in type 1 cells in *Bax*^*−/−*^*Bak*^*−/−*^*Bok*^*−/−*^ embryos and can only be abrogated by the loss of caspase-8. Necroptosis, a lytic form of programmed cell death that is executed by the activation of the pore-forming protein MLKL, is induced when death receptors are stimulated and caspase-8 is absent or inhibited [9]. We examined the impact of the combined absence of both apoptotic pathways and necroptosis on embryonic development.

## Results

**Fig. 1:**
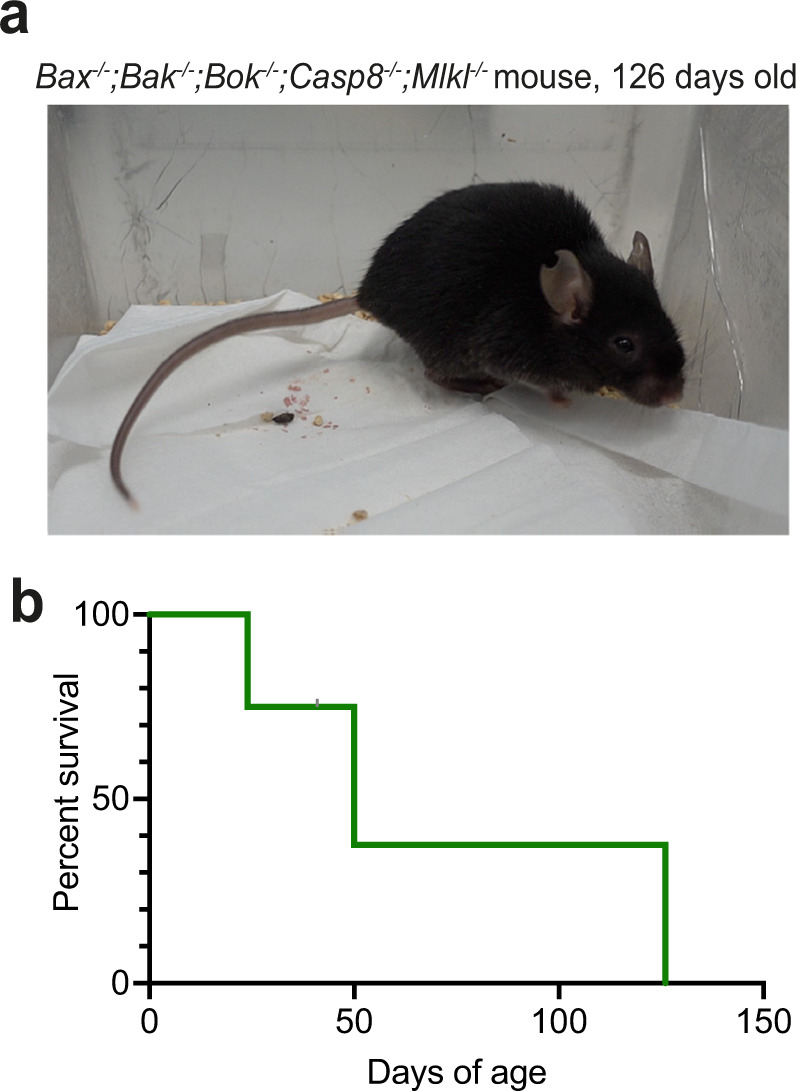
A small number of *Bax*^−/−^*;Bak*^−/−^*;Bok*^−/−^*;Casp8*^−/−^*;Mlkl*^−/−^ quintuple knockout (Q5KO) mice can reach adulthood. **a** Image of the longest surviving *Bax*^*−/−*^*;Bak*^*−/−*^*;Bok*^*−/−*^*;Casp8*^*−/−*^*;Mlkl*^*−/−*^ quintuple knockout mouse at 126 days of age. **b** Survival curve of *Bax*^*−/−*^*;Bak*^*−/−*^*;Bok*^*−/−*^*;Casp8*^*−/−*^*;Mlkl*^*−/−*^ quintuple knockout mice that survived the early postnatal period. *Bax*^*−/−*^*;Bak*^*−/−*^*;Bok*^*−/−*^*;Casp8*^*−/−*^*;Mlkl*^*−/−*^ quintuple knockout mice survived to 50, 24, and 126 days.

The observations that many tissues in which apoptosis was proposed to play a role appear surprisingly normal in *Bax*^*−/−*^*;Bak*^*−/−*^*;Bok*^*−/−*^ embryos suggested that additional programmed cell death pathways might operate alongside the intrinsic apoptotic pathway to allow the shaping of tissues during embryonic and foetal development. To explore this hypothesis, we generated mice that lacked not only the multi-BH (BCL-2 homology) domain effectors of apoptosis, BAX, BAK and BOK, but additionally were also deficient in caspase-8, which is essential for death receptor induced apoptosis [10], and MLKL, which is needed for necroptosis [11]. Note that loss of caspase-8 causes embryonic lethality ~E11.5 due to aberrant necroptosis that can be prevented by concomitant absence of RIPK3 [12, 13] or MLKL [14], which are both essential for necroptosis. For this we crossed *Bax*^*+/−*^*;Bak*^*−/−*^*;Bok*^*−/−*^*;Casp8*^*−/−*^*;Mlkl*^*−/−*^ mice with *Bax*^*+/−*^*;Bak*^*−/−*^*;Bok*^*−/−*^*;Casp8*^*+/−*^*;Mlkl*^*−/−*^ mice offering a 1/8 chance to obtain *Bax*^*−/−*^*;Bak*^*−/−*^*;Bok*^*−/−*^*;Casp8*^*−/−*^*;Mlkl*^*−/−*^ offspring. 151 offspring reached weaning (3 weeks of age) and 147 adulthood (6 weeks of age). Instead of the expected 38, only 3 *Bax*^*−/−*^*;Bak*^*−/−*^*;Bok*^*−/−*^*;Casp8*^*−/−*^*;Mlkl*^*−/−*^ mice reached weaning, two reached adulthood (Table 1). One *Bax*^*−/−*^*;Bak*^*−/−*^*;Bok*^*−/−*^*;Casp8*^*−/−*^*;Mlkl*^*−/−*^ mouse survived for 126 days (Fig. 1a, b). The frequency of *Bax*^*−/−*^*;Bak*^*−/−*^*;Bok*^*−/−*^*;Casp8*^*−/−*^*;Mlkl*^*−/−*^ mice was similar to the frequency of *Bax*^*−/−*^*;Bak*^*−/−*^*;Bok*^*−/−*^ mice at weaning and upon reaching adulthood (3/151 and 2/147 *vs* 7/392 [5] and 4/444 [5], p = 1 and *p* = 0.6, respectively; Fisher’s exact test). Similarly, the frequency of *Bax*^*−/−*^*;Bak*^*−/−*^*;Bok*^*−/−*^*;Casp8*^*−/−*^*;Mlkl*^*−/−*^ mice was similar to *Bax*^*−/−*^*;Bak*^*−/−*^*;Bok*^*−/−*^*;Casp8*^*+/−*^*;Mlkl*^*−/−*^ mice at weaning and upon reaching adulthood (Table 1; *p* = 0.5 and *p* = 1, respectively; Fisher’s exact test). This demonstrates that it is possible to obtain adult mice that lack the intrinsic as well as the death receptor apoptotic pathways and necroptosis. Importantly, the loss of the death receptor apoptotic pathway and necroptosis does not appear to affect the frequency of mice reaching weaning or even adulthood that was previously observed in *Bax*^*−/−*^*;Bak*^*−/−*^*;Bok*^*−/−*^ TKO mice [5].

**Table 1:** *Bax*^*−/−*^*;Bak*^*−/−*^*;Bok*^*−/−*^*;Casp8*^*−/−*^*;Mlkl*^*−/−*^ quintuple knockout (Q5KO) mice are not produced at the expected Mendelian frequency; nevertheless some can reach weaning (~21 days) and even adulthood (≥42 days).

Parental genotypes: Bax^+/−^;Bak^−/−^;Bok^−/−^;Casp8^−/−^;Mlkl^−/−^ x Bax^+/−^ ;Bak^−/−^;Bok^−/−^;Casp8^−/−^;Mlkl^−/−^					
Genotype	Bak^−/−^;Bok^−/−^;Mlkl^−/−^;Casp8^−/−^			Total	*p* value^a^
	Bax^+/+^	Bax^+/−^	Bax^−/−^		
E18.5–E19	13 (15)	37 (30)	10 (15)	60	0.08

Parental genotypes: Bax^+/−^;Bak^−/−^;Bok^−/−^;Casp8^−/−^;Mlkl^−/−^ x Bax^+/−^;Bak^−/−^;Bok^−/−^;Casp8^+/−^;Mlkl^−/−^					
Genotype	Bak^−/−^;Bok^−/−^;Mlkl^−/−^;Casp8^−/−^			Total	*p* value
	Bax^+/+^	Bax^+/−^	Bax^−/−^		
Weaning	41 (38)	107 (76)	3 (38)^b^	151	<10^–6^
Adult	38 (37)	107 (74)	2 (37)^c^	147	<10^–6^

Genotype	Bak^−/−^;Bok^−/−^;Mlkl^−/−^;Casp8^+/^^*−*^			Total	*p* value
	Bax^+/+^	Bax^+/−^	Bax^−/−^		
Weaning	64 (53)	139 (105)	7 (53)^b^	210	<10^–6^
Adult	64 (51)	137 (103)	4 (51)^c^	205	<10^–6^

^a^Observed and expected numbers were compared calculating the cumulative probability distribution of being less or equal to the expected value (pbinom; R version 4.0.5 2021-03-31).

^b,^ ^c^The frequency of *Bax*^*−/−*^*;Bak*^*−/−*^*;Bok*^*−/−*^*;Mlkl*^*−/−*^ animals not significantly different between *Casp8*^*−/−*^ and *Casp8*^*+/−*^ at weaning (^b^, 3 weeks of age, Fisher’s exact test *p* = 0.5) and adulthood (^c^, 6 weeks of age, *p* = 1), but less than expected (*p* value column).

**Fig. 2:**
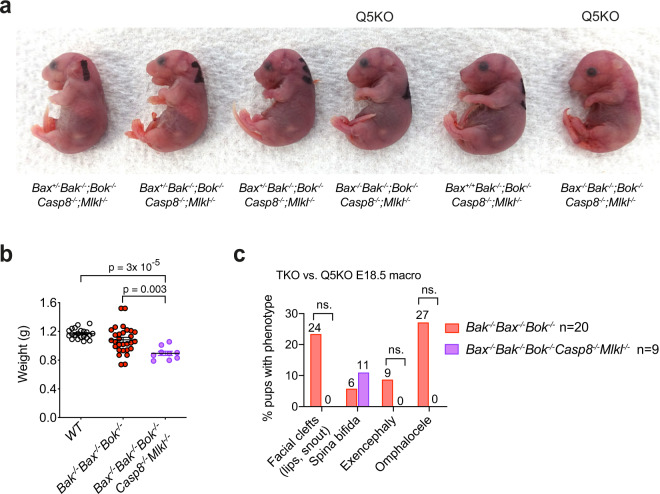
E18.5 *Bax*^*−/−*^*;Bak*^*−/−*^*;Bok*^*−/−*^*;Casp8*^*−/−*^*;Mlkl*^*−/−*^ quintuple knockout (Q5KO) foetuses display a reduction in body weight and externally visible anomalies. **a** Representative image of a litter of E18.5 foetuses of a mating between a *Bax*^+/−^*;Bak*^*−/−*^*;Bok*^*−/−*^*;Casp8*^*−/−*^*;Mlkl*^*−/−*^ female and a *Bax*^+/−^*;Bak*^*−/−*^*;Bok*^*−/−*^*;Casp8*^*−/−*^*;Mlkl*^*−/−*^ male. Offspring genotypes are indicated. **b** Body weights of E18.5 foetuses of the indicated genotypes. Data are presented as mean ± SEM; *p*, one-way ANOVA followed by multiple comparison and Tukey correction for multiple testing. Each dot represents an individual foetus. **c** Percentages of E18.5 *Bax*^*−/−*^*;Bak*^*−/−*^*;Bok*^*−/−*^*;Casp8*^*−/−*^*;Mlkl*^*−/−*^ quintuple knockout and *Bax*^*−/−*^*;Bak*^*−/−*^*;Bok*^*−/−*^ triple knockout foetuses exhibiting externally visible developmental defects (numbers above bars represent the percentages of animals) compared by Fisher’s exact test.

**Fig. 3:**
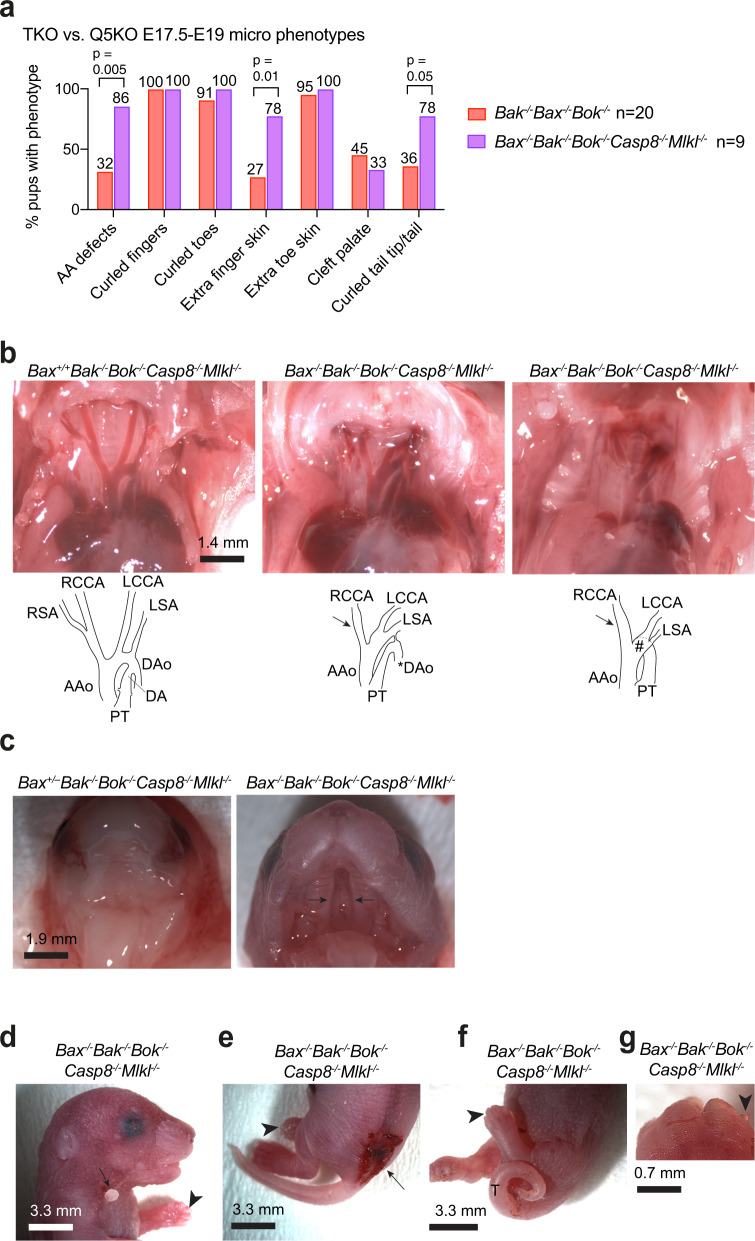
E18.5 *Bax*^*−/−*^*;Bak*^*−/−*^*;Bok*^*−/−*^*;Casp8*^*−/−*^*;Mlkl*^*−/−*^ quintuple knockout (Q5KO) foetuses showed a range of anomalies when examined under the dissection microscope. **a** Percentages of 9 E18.5 *Bax*^*−/−*^*;Bak*^*−/−*^*;Bok*^*−/−*^*;Casp8*^*−/−*^*;Mlkl*^*−/−*^ Q5KO foetuses and 20 E18.5 *Bax*^*−/−*^*;Bak*^*−/−*^*;Bok*^*−/−*^ TKO foetuses exhibiting microscopically visible developmental defects (numbers above bars represent the percentages of animals) compared by Fisher’s exact test. **b** Representative images of the large vessels of an E18.5 *Bax*^+/+^*;Bak*^*−/−*^*;Bok*^*−/−*^*;Casp8*^*−/−*^*;Mlkl*^*−/−*^ control foetus (left panel) and two *Bax*^*−/−*^*;Bak*^*−/−*^*;Bok*^*−/−*^*;Casp8*^*−/−*^*;Mlkl*^*−/−*^ quintuple knockout foetuses. Schematic outlines of the large vessels are shown below the images. The *Bax*^*−/−*^*;Bak*^*−/−*^*;Bok*^*−/−*^*;Casp8*^*−/−*^*;Mlkl*^*−/−*^ quintuple knockout foetus in the middle panel showed the absence of the right subclavian artery (arrow) and an abnormal origin of the descending aorta from the from the pulmonary trunk (*). The *Bax*^*−/−*^*;Bak*^*−/−*^*;Bok*^*−/−*^*;Casp8*^*−/−*^*;Mlkl*^*−/−*^ quintuple knockout foetus in the right panel displayed the absence of the right subclavian artery (arrow) and an absence of the descending aorta. Instead of leading to a descending aorta, the ascending aorta connected abnormally to the pulmonary trunk (#). AAo, ascending aorta; DA ductus arteriosus; DAo descending aorta; PT pulmonary trunk; RSA/LSA right/left subclavian artery; RCCA/LCCA right/left common carotid artery; arrows indicate site where RSA is missing; asterisk indicates DAo with abnormal origin; # indicates abnormal connection between PT and AAo. **c** Representative images of the ventral view of the palate of an E18.5 *Bax*^+/−^*;Bak*^*−/−*^*;Bok*^*−/−*^*;Casp8*^*−/−*^*;Mlkl*^*−/−*^ control foetus (left panel) and a *Bax*^*−/−*^;*Bak*^*−/−*^;*Bok*^*−/−*^;*Casp8*^*−/−*^;*Mlkl*^*−/−*^ quintuple knockout foetus with cleft of the soft and bony palate (arrows). **d** Image of an E18.5 *Bax*^*−/−*^*;Bak*^*−/−*^*;Bok*^*−/−*^*;Casp8*^*−/−*^*;Mlkl*^*−/−*^ quintuple knockout foetus with curled fingers (arrowhead) and protruding skin tissue in the neck region (arrow). **e** Image of an E18.5 *Bax*^*−/−*^*;Bak*^*−/−*^*;Bok*^*−/−*^*;Casp8*^*−/−*^*;Mlkl*^*−/−*^ quintuple knockout foetus with curled toes (arrowhead) and sacral spina bifida (arrow). **f** Image of an E18.5 *Bax*^*−/−*^*;Bak*^*−/−*^*;Bok*^*−/−*^*;Casp8*^*−/−*^*;Mlkl*^*−/−*^ quintuple knockout foetus with curled toes (arrowhead) and curled tail (T). **g** Image of an E18.5 *Bax*^*−/−*^*;Bak*^*−/−*^*;Bok*^*−/−*^*;Casp8*^*−/−*^*;Mlkl*^*−/−*^ quintuple knockout foetus with excess toe tissue (arrowhead).

Most *Bax*^−/−^*; Bak*^−/−^*; Bok*^−/−^ mice present with significant developmental abnormalities at E18.5 [5]. To determine whether the additional lack of death receptor induced apoptosis and necroptosis could further exacerbate these developmental abnormalities we generated E18.5 *Bax*^−/−^*;Bak*^−/−^*;Bok*^−/−^*; Casp8*^−/−^*;Mlkl*^−/−^ foetuses from intercrosses of *Bax*^+/−^*;Bak*^−/−^*;Bok*^−/−^*;Casp8*^−/−^*;Mlkl*^−/−^ mice (Fig. 2a) and compared them to E18.5 *Bax*^−/−^*;Bak*^–/–^*;Bok*^*−/−*^ foetuses. From a total of 60 foetuses harvested, 10 were identified as *Bax*^*−/−*^*;Bak*^*−/−*^*;Bok*^*−/−*^*;Casp8*^*−/−*^*;Mlkl*^*−/−*^ (not significantly different from the expected 15, *p* = 0.08; Table 1). This genotype frequency is similar to the distribution seen for *Bax*^*−/−*^*;Bak*^*−/−*^*;Bok*^*−/−*^ foetuses at E18.5 (10/60 vs 39/193 [5]; *p* = 1, Fisher’s exact test). *Bax*^*−/−*^*;Bak*^*−/−*^*;Bok*^*−/−*^*;Casp8*^*−/−*^*;Mlkl*^*−/−*^ foetuses had a significantly lower body weight compared to the *Bax*^*−/−*^*;Bak*^*−/−*^*;Bok*^*−/−*^ foetuses and wild-type E18.5 foetuses (Fig. 2b). External examination revealed that E18.5 *Bax*^*−/−*^*;Bak*^*−/−*^*;Bok*^*−/−*^*;Casp8*^*−/−*^*;Mlkl*^*−/−*^ foetuses, like *Bax*^*−/−*^*;Bak*^*−/−*^*;Bok*^*−/−*^ foetuses, displayed spina bifida. In contrast, unlike *Bax*^*−/−*^*;Bak*^*−/−*^*;Bok*^*−/−*^ foetuses, *Bax*^*−/−*^*;Bak*^*−/−*^*;Bok*^*−/−*^*;Casp8*^*−/−*^*;Mlkl*^*−/−*^ foetuses did not show external facial clefts, exencephaly or omphalocele, although the differences in the incidence of these defects did not reach statistical significance at the number of animals available (Fig. 2c). The frequency of most defects assessed microscopically were similar between *Bax*^*−/−*^*;Bak*^*−/−*^*;Bok*^*−/−*^*;Casp8*^*−/−*^*;Mlkl*^*−/−*^ *and Bax*^*−/−*^*;Bak*^*−/−*^*;Bok*^*−/−*^ foetuses, although some variation in penetrance was observed (Fig. 3a). Aortic arch (AA) defects (Fig. 3a, b), cleft palate (Fig. 3a, c), curled fingers (Fig. 3a, d), curled toes (Fig. 3a, e, f), curled tails (Fig. 3a, f) and extra toes tissue (Fig. 3a, g) were observed in the *Bax*^*−/−*^*;Bak*^*−/−*^*;Bok*^*−/−*^ E18.5 foetuses. The penetrance of most anomalies was not significantly different between genotypes (Figs. 2c and 3a). While the frequencies of aortic arch defects, extra finger tissue, and curled tail were increased in *Bax*^*−/−*^*;Bak*^*−/−*^*;Bok*^*−/−*^*;Casp8*^*−/−*^*;Mlkl*^*−/−*^ foetuses compared to *Bax*^*−/−*^*;Bak*^*−/−*^*;Bok*^*−/−*^ E18.5 foetuses (Fig. 3a), an increase in penetrance or severity did not apply to the majority of defects universally (Figs. 2c and 3a).

## Discussion

Since in embryos deficient for the intrinsic apoptotic pathway (*Bax*^*−/−*^*;Bak*^*−/−*^*;Bok*^*−/−*^) many tissues that were long thought to require apoptosis for development appeared surprisingly normal and since some of these mice could even reach early adulthood [5], we explored whether foetuses deficient in both intrinsic as well as death receptor induced apoptosis and necroptosis (*Bax*^*−/−*^*;Bak*^*−/−*^*;Bok*^*−/−*^*;Casp8*^*−/−*^*;Mlkl*^*−/−*^) would have more severe abnormalities. Our findings presented here demonstrate that, while the penetrance of the observed defects varied between these two genotypes, overall, the additional absence of death receptor induced apoptosis and necroptosis, due to the loss of caspase-8 and MLKL, had no effect on the survival rate and little effect on the developmental abnormalities caused by the absence of the intrinsic apoptotic pathway (loss of BAX, BAK, and BOK).

Nevertheless, the three defects that were more common in *Bax*^*−/−*^*;Bak*^*−/−*^*;Bok*^*−/−*^*;Casp8*^*−/−*^*;Mlkl*^*−/−*^ than in *Bax*^*−/−*^*;Bak*^*−/−*^*;Bok*^*−/−*^ pups, aortic arch defects, extra finger skin tissue, and curly tails, could indicate some role of the extrinsic apoptotic pathway or necroptosis in restricted tissues. Loss of necroptosis alone [11] or combined absence of death receptor induced apoptosis plus necroptosis do not cause any developmental abnormalities [13, 14]. However, inhibition of the intrinsic apoptotic pathway synergises with defects in the death receptor apoptotic pathway in causing lymphadenopathy [15, 16]. We, therefore, assume that, if any additional defects in embryonic development exist at all, it is the loss of death receptor induced apoptosis rather than the absence of necroptosis that causes an increase in some developmental abnormalities in *Bax*^*−/−*^*;Bak*^*−/−*^*;Bok*^*−/−*^*;Casp8*^*−/−*^*;Mlkl*^*−/−*^ mice. Perhaps some cells that would normally be removed by the intrinsic apoptotic pathway during embryogenesis but cannot be removed in this manner due to the absence of BAX, BAK, and BOK, could be induced to undergo death receptor induced apoptosis instead, with the ligand(s) for the relevant death receptor(s) presumably provided by neighbouring cells. This would mean that these cells must be type 1 cells, since type 2 cells require the BH3-only protein BID as well as BAX and/or BAK for death receptor induced killing (i.e. in type 2 cells the loss of BAX and BAK would already suffice to block death receptor induced apoptosis) [7].

In conclusion, our work shows that, surprisingly, a small number of mice can reach weaning and even adulthood despite the complete absence of the intrinsic as well as the death receptor induced apoptotic pathways and also necroptosis. Perhaps additional cell death pathways, either known ones, such as cell death associated with autophagy [17], or yet to be discovered cell death pathways also play roles alongside apoptosis in embryonic and foetal development.

## Materials and methods

### Mice

All experiments with mice were performed with the approval of the Walter and Eliza Hall Institute Animal Ethics Committee. Animals were handled according to the Australian code of practice for the care and use of animals for scientific purposes.

*Bok*^*−/−*^*;Bax*^*−/−*^*;Bak*^*−/−*^ triple knockout (TKO) mice on a C57BL/6 background have been described previously [5]. *Bax*^*−/−*^*;Bak*^*−/−*^*;Bok*^*−/−*^*;Casp8*^*−/−*^*;Mlkl*^*−/−*^ quintuple knockout (Q5KO) animals were derived by crossing *Bok*^*−/−*^*Bax*^+/−^*Bak*^*−/−*^ mice with *Casp8*^−/−^;*Mlkl*^−/−^ mice [14], also on a C57BL/6 background. Following subsequent rounds of breeding, *Bax*^+/−^*;Bak*^*−/−*^*;Bok*^*−/−*^*;Casp8*^*−/−*^*;Mlkl*^*−/−*^ and *Bax*^*+/−*^*;Bak*^*−/−*^*;Bok*^*−/−*^*;Casp8*^*+/−*^*;Mlkl*^*−/−*^ animals were obtained and were intercrossed to generate *Bax*^*−/−*^*;Bak*^*−/−*^*;Bok*^*−/−*^*;Casp8*^*−/−*^*;Mlkl*^*−/−*^ Q5KO mice.

For timed matings, noon of the day on which the vaginal plug was first observed was defined as embryonic day 0.5 (E0.5). Mouse foetuses were recovered at E18.5–E19 (just before birth) by Caesarean section. Animals were weaned between 19 and 23 days after birth and deemed adults at 42 days of age. Mice were genotyped by PCR as described previously [5, 14]. All mice with a *Casp8*^*−/−*^*;Mlkl*^*−/−*^ genotype, regardless of their genotype for *Bax*, *Bak* and *Bok*, that reached >150 days developed lymphadenopathy, splenomegaly, and/or hepatomegaly, as previously described for *Casp8*^−/−^*;Mlkl*^*−/−*^ mice [10].

### Microscopy

For detailed phenotypic examination, E18.5 foetuses were euthanised by cooling. Dissections were performed using the Stemi 2000-C dissecting microscope. Pups were photographed with a digital camera (AxioCam HR, Carl Zeiss).

### Quantification and statistical analysis

The ratio of offspring obtained from intercrosses of *Bax*^*+/−*^*;Bak*^−/−^*;Bok*^−/−^*;Casp8*^−/−^*;Mlkl*^−/−^ or crosses of *Bax*^+/−^;*Bak*^−/−^*;Bok*^−/−^*;Casp8*^−/−^*;Mlkl*^−/−^ with *Bax*^+/−^;Bak^*−/−*^*;Bok*^−/−^*;Casp8*^+/−^*;Mlkl*^*−/−*^ mice at different stages were analysed calculating the cumulative probability distribution of being less or equal to the expected value (pbinom; R version 4.0.5 2021-03-31). Frequencies of defects in *Bax*^−/−^*;Bak*^−/−^*;Bok*^−/−^*;Casp8*^−/−^*;Mlkl*^−/−^ quintuple knockout and *Bax*^−/−^*;Bak*^−/−^*;Bok*^−/−^ triple knockout were compared by Fisher’s exact test using Stata/SE software16.1 (StataCorp, Texas). Column graphs were prepared using GraphPad Prism software 9.0. The statistical tests used are stated in the figure legend. The number of replicates (*n*) is defined as number of animals stated in Table 1 or in the figure legends.

## Data Availability

The authors declare that all data supporting the findings of this study are available within the article and the Supplementary Materials.
